# Jail-based treatment for opioid use disorder in the era of bail reform: a qualitative study of barriers and facilitators to implementation of a state-wide medication treatment initiative

**DOI:** 10.1186/s13722-022-00313-6

**Published:** 2022-06-02

**Authors:** Noa Krawczyk, Sachini Bandara, Sydney Merritt, Hridika Shah, Alexandra Duncan, Brendan McEntee, Maria Schiff, N. Jia Ahmad, Sara Whaley, Amanda Latimore, Brendan Saloner

**Affiliations:** 1 Center for Opioid Epidemiology and Policy, Department of Population Health, NYU Grossman School of Medicine, 180 Madison Ave, Room 4-12, New York, NY USA; 2 Department of Mental Health, Johns Hopkins Bloomberg School of Public Health, Baltimore, MD USA; 3 Department of Health Policy and Management, Johns Hopkins Bloomberg School of Public Health, Baltimore, MD USA; 4 The Pew Charitable Trusts, Washington, DC USA; 5 Johns Hopkins University School of Medicine, Baltimore, MD USA; 6 Center for Addiction Research and Effective Solutions, American Institutes for Research, Arlington, VA USA

**Keywords:** Jail, Opioid use disorder, Treatment, Re-entry, Criminal legal system, Criminal justice

## Abstract

**Background:**

Until recently, few carceral facilities offered medications for opioid use disorder (MOUD). Although more facilities are adopting MOUD, much remains to be learned about addressing implementation challenges related to expansion of MOUD in carceral settings and linkage to care upon re-entry. This is particularly important in jails, where individuals cycle rapidly in and out of these facilities, especially in jurisdictions beginning to implement bail reform laws (i.e., laws that remove the requirement to pay bail for most individuals). Increasing access to MOUD in these settings is a key unexplored challenge.

**Methods:**

In this qualitative study, we interviewed staff from county jails across New Jersey, a state that has implemented state-wide efforts to increase capacity for MOUD treatment in jails. We analyzed themes related to current practices used to engage individuals in MOUD while in jail and upon re-entry; major challenges to delivering MOUD and re-entry services, particularly under bail reform conditions; and innovative strategies to facilitate delivery of these services.

**Results:**

Jail staff from 11 New Jersey county jails participated in a baseline survey and an in-depth qualitative interview from January–September 2020. Responses revealed that practices for delivering MOUD varied substantially across jails. Primary challenges included jails’ limited resources and highly regulated operations, the chaotic nature of short jail stays, and concerns regarding limited MOUD and resources in the community. Still, jail staff identified multiple facilitators and creative solutions for delivering MOUD in the face of these obstacles, including opportunities brought on by the COVID-19 pandemic.

**Conclusions:**

Despite challenges to the delivery of MOUD, states can make concerted and sustained efforts to support opioid addiction treatment in jails. Increased use of evidence-based clinical guidelines, greater investment in resources, and increased partnerships with health and social service providers can greatly improve reach of treatment and save lives.

**Supplementary information:**

The online version contains supplementary material available at 10.1186/s13722-022-00313-6.

## Background

Largely due to the criminalization of addiction, 70% of people who use heroin in the U.S. encounter the criminal legal system [1]. Correctional settings could therefore be an important touchpoint to link persons with opioid use disorder (OUD) to evidence-based treatment and other services. However, most correctional facilities have historically failed to provide such linkage, with overdose risk recorded to be up to 100 times greater than among the general population in the weeks following release from incarceration [2–5]. This higher overdose risk arises in large part from loss of tolerance and a lack of available maintenance treatments for OUD in correctional facilities. Until recently, stigma against medications for opioid use disorder (MOUD) and a lack of infrastructure and resources to deliver such treatments resulted in few correctional facilities offering MOUD to the majority of individuals with OUD [6–8]. In addition, less than 5% of persons referred to treatment in the community by the criminal legal system receive MOUD [9]. Years of public health advocacy around this issue [10], along with several lawsuits against correctional facilities that refused to provide MOUD in the midst of a worsening opioid epidemic [11], have pushed jurisdictions around the country to increase access to MOUD in correctional settings. Despite initial success in some model states such as Rhode Island [12] much remains to be learned about addressing implementation challenges related to acceptance, adoption and sustainability of MOUD behind bars and upon release [13].

Local jails experience particular challenges to implementing MOUD programs [13]. Unlike prisons, jails are typically overseen by local governments and primarily house persons awaiting trial who cycle rapidly through them, often with unpredictable lengths of stay. New Jersey is one state which, against the backdrop of a worsening overdose epidemic [14], has been working to expand access to MOUD. While all state prisons in New Jersey already offer MOUD, most county jails have historically lacked such treatments, motivating efforts to support the expansion of MOUD in jails. A recent statewide effort in 2019 led and funded by the New Jersey Department of Human Services, Division of Mental Health and Addiction Services in collaboration with the New Jersey Departments of Corrections and Health, aimed to provide financial support and technical assistance to support jails in: (1) expanding jail MOUD treatment and re-entry services, and; (2) collecting statewide data to track the adoption and implementation of MOUD in county jails. While there were no standardized guidelines for implementing MOUD across jails, each jail was required to submit its MOUD implementation plan to New Jersey state agencies and report standard outcome metrics to the New Jersey Division of Mental Health and Addiction Services.

An important factor in the rollout of this effort is that New Jersey recently underwent a large-scale bail reform known as the New Jersey Criminal Justice Reform Act. Passed in 2014 and implemented in January of 2017, this act eliminated cash bail options throughout the state, leaving judges to determine whether to release or detain someone prior to trial based on a standardized risk assessment, without consideration of their ability to pay bail [15]. Such bail reform policies significantly reduce the length of detention for most individuals who enter jail, who would have otherwise remained behind bars awaiting trial if they were unable to raise the bail [16]. Some version of bail reform has already been passed in seven states [17] and it is a component of President Biden’s Justice Agenda [18]. Bail reform is a critical step in decreasing the volume of persons detained without trial and reducing inequities in the disproportionate incarceration of racial and ethnic minorities and those who cannot afford to pay bail [19]. The Criminal Justice Reform in New Jersey already led to the successful reduction in the jail population and significantly shortened lengths of stay, from an average 7.4 days in 2014 to 3.7 days in 2017 [20].

Against the backdrop of bail reform, New Jersey’s experience has raised questions about the best practices for implementing MOUD programs that cater to individuals who enter jail for very brief periods of time. This challenge has been further complicated since 2020 by the COVID-19 pandemic and rapid efforts to manage the spread of disease. In the current qualitative study, our aims were threefold: (1) To characterize the current capacity and range of MOUD services being offered to individuals residing in New Jersey jails and re-entering the community; (2) To assess major challenges to delivering MOUD and re-entry services in jails, especially under bail reform conditions; and (3) To describe innovative solutions and strategies that jails have implemented to facilitate delivery of these services. Learning from the experience of New Jersey may help other regions that are aiming to push for expansion of access to MOUD in local jails.

## Methods

Reporting of the qualitative research process followed the Consolidated criteria for reporting qualitative research (COREQ) [21]. The COREQ checklist is available in Additional file 1: Appendix S1.

### Sample

The study focused on county jails in New Jersey. All 21 New Jersey counties were invited to participate in a baseline jail survey fielded in October–November 2019 followed by in-depth interviews. 17 jails responded to the survey during the study period and consented to use of the survey for research purposes, out of which 11 conducted a follow up interview. Survey and interview respondents included jail staff who could address OUD treatment and re-entry practices, such as a warden, medical director, and re-entry coordinator. Participation was voluntary but encouraged by the New Jersey County Jail Wardens Association as well as the New Jersey Division of Mental Health and Addiction Services. Data were collected to better tailor technical assistance efforts to jails’ needs and identify existing barriers and facilitators to implementing MOUD. In this paper, we report findings from the 11 jails that completed both the baseline survey during the initial data collection period and a follow-up in-depth interview.

### Baseline survey

Invitations for baseline surveys were sent out by email via the president of the New Jersey Jail Wardens Association and by the study authors. Jail staff had the option of completing the survey electronically or over the phone. Survey domains included: current jail census and estimated prevalence of OUD, OUD screening and data collection practices, management and operation of medical services within the jail, availability and eligibility for MOUD provided in jail, and re-entry practices related to linking individuals to OUD treatment and other services at release.

### Qualitative interviews

Following completion of the baseline survey, all jails were invited via email to participate in an in-depth, semi-structured interview. A semi-structured interview guide was developed and partially tailored to each jail based on their response to the survey, intending to address three domains of interest (1) Current capacity and practices for delivering MOUD services in the jail and at re-entry; (2) Primary challenges to delivering MOUDs services, particularly in the context of bail reform; (3) Strategies that have facilitated delivery of these services. For each interview, it was requested that at least one member of jail leadership participate (e.g. Jail Warden) and one member from the medical team participate (e.g. Jail Medical Director). Interviewers on the study team (NK, SB, SM, NJM, AD, MS) had either doctoral or masters’ level training in research and conduct of interviews and focus groups, and no prior relationship with study participants. Other members of the technical assistance team were also invited to participate in the calls (BM, SW, AL). Interviews began in January–March 2020, and after six interviews, were paused due to the immediate emergence of the COVID-19 crisis. Recruitment for interviews resumed from June to September of 2020, after which a semi-structured interview question was added regarding the impact that COVID-19 has had on MOUD operations and re-entry efforts. Interviews lasted approximately 60 min and were conducted via video and/or phone calls, recorded and transcribed. Notes were taken manually for 1 jail that did not allow recording. After 11 jail interviews were completed, the study team decided to halt continuous recruitment for qualitative interviews due to ongoing hurdles due to the COVID-19 pandemic and consensus that thematic saturation was reached. Information on the 6 jails that completed a baseline survey but did not complete an interview is presented in Additional file 2: Appendix S2.

### Analysis

Summary statistics were generated from baseline surveys. Transcribed interviews were uploaded to Dedoose [22], a collaborative web application for managing, analyzing and presenting qualitative and mixed methods research data across multiple users. Interview transcripts were analyzed using a staged hybrid inductive–deductive thematic analysis approach [23]. The research team created an initial codebook based on study questions of interest, a priori knowledge on the subject and summary notes from the interviews. Four members of the study team (NK, SB, SM, NJM) piloted the codebook by double-coding four transcripts. Afterwards, our team met to discuss initial themes that arose in interviews and note any additional codes or differences in interpretation of codes and revise the codebook as needed. The goal was not to measure exact agreement, but to discover concepts and themes that would reveal content for discussion of our main study questions of interest [24]. After these discussions, the remaining seven interviews were coded by a single study team member using the final codebook. After completion of coding, the study team reviewed content and analyzed narratives using thematic analysis [25] to identify primary themes and subthemes that arose related to the research questions. Common sentiments were paraphrased and illustrative quotes were selected and presented to exemplify primary study themes. The [*Johns Hopkins Bloomberg School of Public Health*] Institutional Review Board approved this study.

## Results

### Characteristics of participating jails

We include summary statistics from 11 counties that responded to our baseline jail survey and conducted a qualitative interview (Table 1). Jails reported an average of 5982 non-unique admissions in 2019, ranging from 1610 to 11,067, and an average 5519 non-unique releases, ranging from 1594 to 8914. This indicates an overall increase in volume of persons detained in New Jersey jails in 2019. Jails were also asked to report the number of persons detained on a specific date in September, which led to an overall a mean of 640 persons, ranging from 194 to 2463. Jails were asked to provide their best estimates for prevalence of OUD among their detained population based on existing assessments used to screen for OUD, which was on average 23% (ranging 8–49%). Three quarters of jails used an electronic recording system for provision of MOUD medical services. Table 1 displays a breakdown of available re-entry services across jails: 91% of jails had dedicated discharge staff, 36% provided naloxone upon release, 73% made medical appointments in the community, 64% helped reactivate Medicaid at release and 36% had seperate protocols for individuals staying less than vs. more than 48 h.

**Table 1:** Characteristics of jails included in sample (N = 11), reporting for year 2019

Characteristics of jail population, 2019	Mean	Range
Number of yearly non-unique admissions	5982	1610 –11,067
Number of yearly non-unique releases	5519	1594–8914
Number detained on single day September 30, 2019	640	194–2463
Percent of detained with OUD on September 30, 2019 (N = 8)	23	8–49

Characteristics of jail medical records and re-entry services	Number	Percent (%)
Have electronic medical record system	8	73
Re-entry services offered for individuals with OUD		
Have dedicated discharge staff	10	91
Provide naloxone at release	4	36
Make medical appointments in community at discharge	8	73
Help reactivate Medicaid at release	7	64
Have different protocols for individuals staying 48 h or less	4	36

SOURCE: Authors’ analysis of jail survey data. Only 8 jails responded to the question on percent of detained with OUD. All estimates were based on self-report from jail leadership/staff

**Fig. 1:**
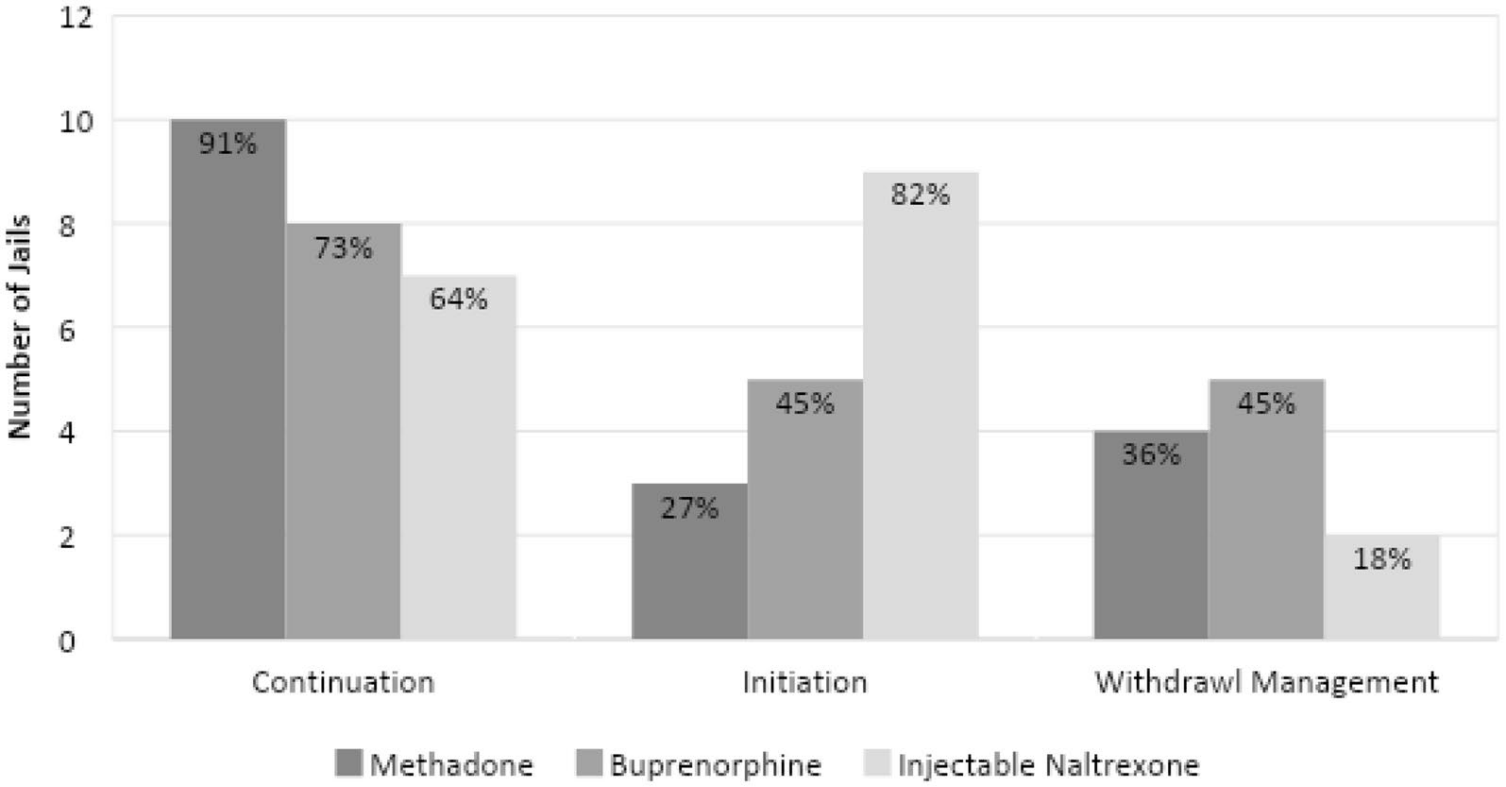
MOUD services available at county jails as reported by jail leadership

Figure 1 presents the MOUD services reported by the 11 jails, disaggregated by medications used and whether services were available for those who already were in treatment prior to jail entry, for new initiates, or for withdrawal management. Most (91%) of the jails reported continuing methadone for those who were already receiving treatment in the community, 73% reported continuing buprenorphine and 64% reported continuing injectable naltrexone. Only 27% were prepared to initiate methadone treatment for those who were treatment naïve at admission, 45% were prepared to initiate buprenorphine, and most (82%) were prepared to initiate injectable naltrexone. 36%, 45% and 18% of jails respectively reported that they also used methadone, buprenorphine and injectable naltrexone for purposes of withdrawal management.

### Themes from jail staff interviews

Our qualitative thematic analysis of interviews with jail staff revealed six overarching themes related to the provision of MOUD and re-entry services in New Jersey jails.

## Theme 1: Practices for delivering MOUD vary drastically across county jails

Jail staff indicated a wide range of practices for delivering MOUD behind bars and connecting individuals with MOUD services at re-entry. Most jails reported a unique set of criteria to determine eligibility to receive MOUD during incarceration, how and when individuals were screened for eligibility, and which medications were available for initiation of MOUD, continued maintenance, or withdrawal management. Jail staff described that practices for determining eligibility and the process by which MOUDs were delivered often depended on preferences of medical directors or administrators. Jails often held clinical protocols based on what medication was perceived as most convenient or effective by the jail’s medical personnel, rather than a set of evidence-based clinical guidelines. For example, one respondent indicated their jail’s preference for extended-release naltrexone, which required all individuals with OUD, regardless of preference or medical need, to go through the withdrawal management protocol at admission:

"*Typically speaking, when they come in they will go to a 5-day detoxification program, and the reason for that is to preserve the opportunity to start them on extended-release naltrexone. Nothing prohibits us from starting them on one of the [opioids] either full or partial opioid agonists, but we do detox everybody to abstinence and then start the process at approximately day 5.*"

Some jails also reported withdrawal management (often described as detox or tapering protocols) using adjunctive medications such as Clonidine rather than first-line MOUD treatments, even if individuals were already taking prescribed, FDA-approved medications like buprenorphine as part of their addiction treatment plan prior to incarceration. This is in spite of current guidelines for MOUD suggesting buprenorphine or methadone can be administered within hours of having used opioids and that MOUDs should be selected based on individual patient preference and circumstances [26]. Similarly, there was a variety of practices and expectations regarding what types of psychosocial services (e.g. counselling, support groups) were available or required for individuals receiving MOUD. While many staff believed psychosocial services were beneficial when available, some jails mandated such services for individuals receiving MOUD. As one respondent explained, talk therapy was considered an essential component of their jail’s treatment regimen for individuals initiating MOUD (referred to as MAT or “Medication Assisted Treatment” below):

*“MAT itself as a singular act is a pointless gesture without the talk therapy to go along with it.”*

## Theme 2: Limited resources and highly regulated operations challenge MOUD delivery

When jail staff were asked about primary challenges they experienced in operating and expanding access to MOUD treatment in jails, many discussed the limited funding and resources to sustain such operations. As one respondent described:

*“We all know what the elephant in the room is. It’s funding. That’s the biggest challenge. If we had the funding, we could certainly expand our program.”*

Some respondents explained that delivery of MOUD requires heavy use of staff time as it involves regulated movement of people in an environment that’s already highly structured and rigid. This challenge was especially noted as a burden to providing methadone treatment. Due to strict federal regulations for provision of methadone that would require jails to hold a license from the Drug Enforcement Administration to provide methadone treatment internally [27], multiple jails instead partnered with external opioid treatment programs to administer methadone treatment. For some jails, this meant transporting individuals out to an external clinic on a daily or near daily basis. For others, the jail staff picked up doses of methadone from community OTPs to administer to individuals in jail. One respondent described the burden of transporting patients regularly to methadone clinics, and how decreasing the frequency of these visits would significantly alleviate time and resources:

*“They probably would like to have an extension in terms of how many days worth of methadone could be held at the facility… So if there was more flexibility in terms of how many days the patient has to return to the outside center, that would be a big—that would be a big hurdle that would be overcome.”*

Respondents further described that even internal provision of medications could be burdensome and resource intensive. One respondent described the process involved in delivering buprenorphine (referred to by its brand name “Suboxone”), and how this was further slowed by efforts to minimize its diversion, often cited as a concern for offering this medication in jail settings [28, 29].

*“Right now, I have about close to 40 patients on Suboxone alone. To transfer all 40 patients to us in the clinic to give them the Suboxone, and the timing for the nurses and the custody staff, it takes us almost about 3 h to medicate those patients because not only are we giving them the medication, we are also observant and taking it for side effects of it, making sure that they take it also because sometimes patients may try to divert the medication.”*

## Theme 3: Short jail stays create slim window to provide MOUD services

Another common challenge discussed in delivering MOUD in jails and linking people to community-based care at re-entry was the brief time that many individuals spent in jail and the unpredictable timing of entry and release. This was perceived to have become especially pertinent following New Jersey’s bail reform effort, which significantly shortened the average length of stay [20]. While multiple respondents credited this reform for lowering the volume of persons detained in recent years, they also explained that this made efforts to manage or initiate MOUD extremely challenging. As one respondent described:

*"I think one of the barriers to our success is the fact of bail reform. They’re in and out before we can safely manage their withdrawing from poly substance [use] and getting them initiated on a program that would involve pharmacological interventions. So I think that would be our greatest asset, would be the early identification, and one of the barriers is the inability to get them initiated on an appropriate clinical program."*

Some staff discussed that despite an overall lower volume of individuals detained following bail reform, the constant movement of people in and out of jail and the re-incarceration of the most complex patients nonetheless made jail operations difficult to manage:

*“Our population was at one point was up around 1300. We’re down around 550 as we speak. And you’d think that would make life easier, but it seems like we have 500 of the most challenging patients in the community.”*

For some, the brief time from admission to release was denoted as the main reason for not initiating certain MOUD behind bars. One respondent explained how their jail was not planning on offering buprenorphine due to the nature of such short stays:

*“We don’t intend to start Suboxone here or Buprenorphine here. Because of number one, time constraints, and number two-time constraints, I mean, the average stay of the [inaudible] will probably be 3 days. It’s because they come in and out which is different from the prison system.”*

Some respondents mentioned that given unpredictable timing of discharge, it was ineffective to allocate services based on an individual’s planned release date. As one respondent noted:

*“Those jails that attempt to time their clinical treatment based on what they perceive might be the patient’s time of release or time remaining are just fraught with problems and you end up losing a substantial number of the patients if that’s your strategy.”*

## Theme 4: Limited MOUD and resources in the community challenge linkage to care at re-entry

For most jails, re-entry support and linkage to community-based MOUD treatment were considered a central component of their MOUD programs. When asked about challenges to coordinating re-entry services, many mentioned a lack of available community-based MOUD providers with whom they could link individuals upon discharge. As one respondent commented:

*“Let’s say if they get discharged after 4 or 5 days, there’s really no place for them to go because of the availability of resources outside. Because I cannot just call any physician there and say, “Can you take care of this patient?“ That won’t probably work up here.”*

The perceived lack of available MOUD providers in the community was not only a problem for discharge planning but also influenced whether medical staff were comfortable treating individuals in jails. Others also added that beyond a shortage of community-based MOUD providers, released individuals experienced additional challenges. Some individuals whose Medicaid was suspended upon incarceration are unaware of when their benefits would be reactivated upon release. Many find themselves back in the community without engaging in health services because of perceived gaps in insurance coverage. This is exacerbated by multiple other socioeconomic challenges that make it extremely difficult to engage and remain in care as they return to the community. As one respondent described:

*“Some of the barriers I would say is lack of funding, lack of resources, lack of housing for individuals. A lot of time, if they’re homeless, it makes it difficult to link them to such services because they don’t have a mailing address and/or a steady address that a treatment provider can come pick them up for [inaudible] program or partial care program.”*

## Theme 5: Facilitators and creative solutions for delivering MOUD in the face of challenges

Despite numerous hurdles and challenges to providing MOUD, some jails were committed to overcoming obstacles to address the needs of individuals with OUD. One area in which jails showed significant creativity in adapting to the post-bail reform conditions was in finding ways to expedite the intake, initiation and linkage process for individuals that had short stays in jail. One respondent described their intake process for MOUD:

*“We typically can start medications very, very quickly. We don’t really have a good grasp of how long any specific person’s going to remain here. So we start medication as soon as they are clinically qualified. We don’t use jail times, transfer dates, time close to release, as prerequisites for treatment.”*

Some described incorporating screening tools for OUD treatment earlier on in the jail intake process, and even beginning to plan the discharge and re-entry process as soon as individuals entered the jail. As one respondent noted:

*“Discharge planning starts from pretty much the time they walk through the door.”*

Another strategy employed to help facilitate linkage to community-based care for those on buprenorphine was to provide bridge prescriptions paid for by the jail to serve as a buffer between jail release and entry into a community based-program. As a respondent described:

*“Upon discharge, we first [send] over a prescription to the pharmacy up to 3 days. The patient has 3 days to pick it up. We give them the address and the name of the pharmacy. And they show their IDs. And they pick the medication up for 3e days. So that it will give them enough time to make appointments to go and see the outside provider.”*

Flexibility regarding length of the bridge prescriptions varied across jails, from a 3-day prescription as described above, up to 14-day bridge prescriptions. Still, some jail providers were less comfortable providing bridge prescriptions, especially when individuals did not have a specific community-based provider already identified:

*“[The doctor’s] only concern is to ensure that they’re obviously already linked up with a provider on the outside. Otherwise he would be uncomfortable with allowing them to leave with a prescription.*

Multiple jails established partnerships with community-based clinics and organizations to help coordinate MOUD services behind bars and at re-entry. Some described partnering with local opioid treatment programs to provide expedited methadone doses to individuals who are detained. Others mentioned the utility of partnering with re-entry organizations to help coordinate services for individuals upon release, including help with Medicaid reactivation and other health and social needs. One respondent described:

*“We have just a wealth of re-entry folks that come in every week and we meet and we talk about discharge planning needs, re-entry needs. I mean that can range from mental health to addictions to medical to housing to education, GED, I mean we could really go on and on.”*

Finally, some jails exhibited creativity in addressing concerns around buprenorphine diversion, which was often described as a resource- and operational burden of providing MOUD behind bars. One respondent described a person-centered approach to how their jail addressed concerns around diversion through education and working to understand the root of the problem:

*“When somebody does or there’s an incident of diversion we have a small team that goes and talks to them to figure out what was happening, what caused the diversion. We try and just take away any of the factors that aided in their diversion but also really understand why it was happening and if there was something that we could do to alleviate one of the things that was causing it. We try and just work with the individual to see how we can best help them make a different decision in the future….we’re always looking for solutions to that problem. And a solution is not removing someone from the program. Education, education, and re-education seems to work.”*

## COVID-19 brings on new challenges and potential opportunities

A final theme identified in our interviews related to the changing circumstances affecting the provision of MOUD in jails brought on by the COVID-19 pandemic. For most jails, the pandemic introduced an unprecedented set of challenges to an already resource- and time-constrained environment. Staff expressed that in many ways, the pandemic halted or slowed down MOUD operations and plans to expand MOUD services in 2020. One respondent expressed:

*“We were a couple weeks away in March [2020] when we had our plan in place. COVID hit, and we actually backed-off from implementing the distribution and administration of Suboxone. So right now, we’re on a holding pattern.”*

Some respondents explained how COVID-19 and related closures generated problems for their discharge planning and re-entry processes. As one respondent explained:

*“We used to be able to call our records department, verify court dates for our patients, and kind of have an idea when people were leaving so we could coordinate our discharge planning sessions with them and really try to firm up a plan. Those dates are not always showing in the system because the court is still working almost completely remotely. And so we’ll think people have court, court got cancelled. We’ll have no idea somebody has court, they end up having court and get released.”*

Despite these challenges, some jail staff acknowledged that COVID-19 also allowed for some innovations in delivering OUD treatment and linking individuals to community-based care. For example, one respondent discussed how due to COVID-19 emergency regulations that allowed for use of telehealth to initiate buprenorphine [30], the jail was able to team with an external provider to prescribe buprenorphine via telehealth for individuals that were being released from jail:

*“We had [Omitted organization name] reach out and offer kind of telehealth Suboxone initiation and dosing until people can kind of get their own community provider.”*

Others mentioned how novel uptake of telehealth behind bars could potentially alleviate challenges currently faced around transporting individuals behind bars and coordinating their OUD care. For example, one respondent described how telehealth was being used to deliver psychosocial services:

*“We can still do individual counseling. We’re doing everything on telehealth right now”*

## Discussion

The need to improve access to MOUD in the criminal legal system has been a central topic of discussion in national efforts to address the opioid crisis [31]. Still, little research has described implementation barriers and effective strategies to address them, especially in jail settings that are often decentralized and characterized by unpredictable lengths of stay. We attempted to fill this gap by exploring the experience of 11 New Jersey county jails, each with their own leadership, medical providers and practices, and understand how they are adapting to address the common challenge of increasing access to treatment for individuals with OUD.

The New Jersey initiative to support expansion of jail-based MOUD programs via grant funding provides an example of how states can provide incentives to increase access to evidence-based treatments in county jails. Still, findings demonstrate that the unique circumstances within each jail create vastly different capacity and approaches for delivering care. These differences were evident through a wide array of practices used to determine for whom medications were made available, what requirements and processes were in place for delivery of MOUD and linkage to community services, and even how MOUD services were recorded and tracked.

Findings regarding lack of standardization of MOUD practices, and in some cases, clinical care that was not aligned with best practices, are concerning. Stigma and misconceptions around OUD care [32, 33] persisted, which often led to clinically inappropriate care. This was exemplified by some jails’ endorsement of extended-release naltrexone as a withdrawal medication despite this medication not being clinically indicated as such, certain providers’ preference for naltrexone over opioid agonists despite their stronger evidence-base [34], strict regimens for withdrawal management prior to MOUD initiation, mandates around counselling, and the belief that MOUD was not effective without added behavioral therapies despite evidence to the contrary [35, 36], concerns about allowing individuals to leave the jail with buprenorphine prescriptions, and limited provision of naloxone at jail release. These practices underscore the need to establish a set of regularly updated clinical standards for all jails to follow, and processes by which jails could be held accountable to these standards. This could be coupled with educational efforts to reduce stigma and misperceptions around MOUD safety and effectiveness via partnerships with addiction experts [37].

Jail staff working to expand access to MOUD identified three primary sets of barriers. First, were barriers related to the resource-intensive nature of MOUD operations. Even with help from the State’s new grant program, many jails stated having inadequate funds to support the necessary staff time and space. Second, were barriers related to the short and unpredictable nature of jail stays that became more pertinent following bail reform, and for which staff felt they did not have sufficient time to effectively begin treatment or develop discharge plans. Third, were barriers related to the perceived lack of resources in the community, which limited jails’ ability to transition individuals on MOUD to care upon discharge.

Despite these obstacles, our interviews highlight that many jails are making active efforts to address many of these issues: This included establishing processes for individuals to go through intake and discharge in a timelier manner, forming partnerships with local health providers and re-entry organizations, providing bridge buprenorphine prescriptions, and working to address the root of problems like buprenorphine diversion. During COVID-19, one jail even facilitated telehealth buprenorphine appointments with community-providers prior to release. Still, many barriers identified require greater local and federal policy responses. There remains a clear need for additional training and funding to ensure jails can deliver effective MOUD services. This includes supporting programs that can efficiently deliver MOUD for people entering and leaving jails around the clock. The success of some New Jersey jails and those of other jurisdictions like Philadelphia and Denver [38, 39] in delivering comprehensive jail-based MOUD services may be used as a basis for adapting effective strategies.

Creating learning collaboratives so jail leadership and staff can learn from the experience of others, while removing requirements that are not supported by clinical evidence, such as detoxification protocols and mandated counseling, may allow jails to make better use of existing resources. Still, additional resources to support operations are needed, including funding and training to support better data collection and management, as over a quarter of jails relied on paper records to track OUD-related metrics. Assessing long term outcomes of these programs will also require efforts to link data to health outcomes and service utilization data once individuals are released from incarceration. Federal funding to address the opioid crisis can be used to contribute resources locally to these efforts.

Our findings also suggest that, while challenging, multiple jails were able to create procedures that allowed for rapid MOUD initiation and linkage to services for individuals with very short lengths of stay under bail reform conditions. Efforts to reduce the length of time of incarceration may benefit from complementary efforts to reduce jail volume and turnover of individuals with substance use disorders and other behavioral health needs. Increasing the role of pre-arrest diversion programs that provide an alternative avenue to connect individuals with services [40] and efforts to de-criminalize drug use [41] may additionally help reduce burden in jails. In addition, increasing avenues into community-based and low-barrier treatments outside the criminal legal system remain critical for engaging vulnerable populations [42].

Many respondents expressed a concern for the lack of community preparedness for individuals leaving jails. While New Jersey has an MOUD provider capacity greater than the national average [33], it has one of the lowest rates of MOUD treatment utilization relative to its overdose rate [34]. This highlights the need for continued efforts to expand MOUD treatment capacity in areas that are lacking in MOUD providers and programs, help connect jail staff to existing programs and providers where MOUD is available, and particularly ensure access to low threshold programs that can cater to high needs populations [43]. Long term efforts to remove the X waiver requirement from buprenorphine providers [44], make permanent the recent changes instigated by COVID-19 public health emergency declaration to allow for telephone-based initiation of buprenorphine [45, 46] and expanding access to methadone treatment by allowing for methadone dispensing in community-based pharmacies as recently proposed by new legislation [47] could also be impactful in facilitating treatment continuity for individuals leaving incarceration.

Lastly, activating insurance coverage the day of release for individuals leaving jails is critical for continuity of MOUD. While most states now suspend rather than terminate Medicaid for individuals who are incarcerated in jails [48], reactivation of Medicaid upon release often varies by individual jail and may depend on jails’ relationship with local Medicaid offices and capacity to share data on incarceration status in real time. Allowing Medicaid reimbursement for services provided during incarceration is an additional step forward that would reduce the cost burden on jails and facilitate care continuity [49]. The Medicaid Reentry Act reintroduced in the 117th Congress does just that by proposing Medicaid coverage in the 30 days prelease. Further, multiple states hope to bypass legislation by obtaining section 1115 demonstration waiver approval from the Centers for Medicare and Medicaid Services (CMS) to allow Medicaid reimbursement during incarceration. Multiple states have submitted waivers; all for which CMS’ decision is still pending as of March 2022 [50].

### Limitations

This study has several limitations. First, jails voluntarily chose to participate in the baseline survey and qualitative interviews, and therefore findings may not represent conditions or perceptions across the full set of jails in New Jersey. In particular, jails that did not participate in qualitative surveys had smaller jail populations (average persons detained on single day 262 vs. 640), were less likely to have dedicated discharge staff (50% vs. 73%), and were less likely to initiate methadone (0% vs. 27%) and buprenorphine (33% vs. 45%) at the time of the survey (Additional file 2: Appendix S2), indicating potential additional unexplored barriers in jails that were not included in the qualitative analysis. Second, we surveyed and interviewed jail wardens, contracted or county employed medical staff, and re-entry personnel who self-reported on services offered and jail procedures and we were unable to verify specific operations. Data points such as OUD prevalence were based on self-report by jail leadership and may be unreliable. Views of individuals incarcerated, and their experiences in relation to MOUD access, were not represented, and further research is needed to explore these perspectives. Third, the COVID-19 pandemic emerged as this study was already underway; thus, jails interviewed in later months were operating under different circumstances than those interviewed earlier on in the study. Still, we attempted to address changes related to COVID-19 and how this impacted MOUD efforts in our later interviews. Lastly, results may reflect the unique circumstances of New Jersey, a Northeastern state that already offers MOUD across its state prison system and has undergone particularly substantive bail reform; while these findings may not be directly generalizable to other states or jurisdictions, we aimed to focus on themes and lessons that could apply more broadly to efforts to expand MOUD across correctional facilities and that could be adapted to local circumstances.

## Conclusions

Our study provides an in-depth exploration of one state’s effort to expand MOUD across its county jails. Findings highlight that despite many gaps that remain to be addressed in delivery of effective OUD treatment, states can make concerted efforts to support uptake of MOUD treatment in their criminal legal systems and make sustained efforts to support their improvement. More work is needed to understand how to best address barriers to MOUD in correctional facilities and learn from successful models. It is likely that there might not be a “one size fit all” solution to expanding MOUD in jails, but uptake of evidence-based guidelines, sufficient resources, and better partnerships with health and social service providers will greatly improve reach of effective treatment, reducing the cycle of re-incarceration and saving lives.

## Supplementary Information

**Addtional file 1: Appendix S1.** COREQ (COnsolidated criteria for REporting Qualitative research) Checklist.

**Addtional file 2: Appendix S2.** Characteristics of 6 New Jersey jails that participated in baseline survey and not qualitative interviews.

## Data Availability

All data generated or analysed during this study are included in this published article [and its additional information files].
