# Supplementary material for: Jail-based treatment for opioid use disorder in the era of bail reform: a qualitative study of barriers and facilitators to implementation of a state-wide medication treatment initiative
Source: Addict Sci Clin Pract. 2022 Jun 2;17:30. doi: 10.1186/s13722-022-00313-6 (PMC9161649; doi:10.1186/s13722-022-00313-6)
Supplement: Supplementary file 2 — Addtional file 2: Appendix S2. Characteristics of 6 New Jersey jails that participated in baseline survey and not qualitative interviews. [file 13722_2022_313_MOESM2_ESM.docx]

**Appendix S2: Characteristics of 6 New Jersey jails that participated in baseline survey and not qualitative interviews.**

Table S1: Characteristics of Jails (N=6), Reporting for year 2019

| **Characteristics of Jail Population, 2019** | **Mean** | **Range** |
| --- | --- | --- |
| Number of yearly non-unique admissions (N=4) | 2,240 | 870-3,967 |
| Number of yearly non-unique releases (N=5) | 3,002 | 804-6,298 |
| Number detained on single day September 30, 2019 (N=3) | 262 | 58-407 |
| Percent of detained with OUD on September 30, 2019 (N=0) | N/A | N/A |
| **Characteristics of Jail Medical Records and Re-Entry Services (N=6)** | **Number** | **Percent** |
| Have electronic medical record system | 4 | 67% |
| Re-entry Services Offered for Individuals with OUD |  |  |
| Have dedicated discharge staff | 3 | 50% |
| Provide naloxone at release | 1 | 17% |
| Make medical appointments in community at discharge | 4 | 67% |
| Help reactivate Medicaid at release | 3 | 50% |
| Have different protocols for individuals staying 48 hours or less | 0 | 0% |
| SOURCE Authors’ analysis of jail survey data. | | |

Figure S1: MOUD Services Available at County Jails as Reported by Jail Leadership

*
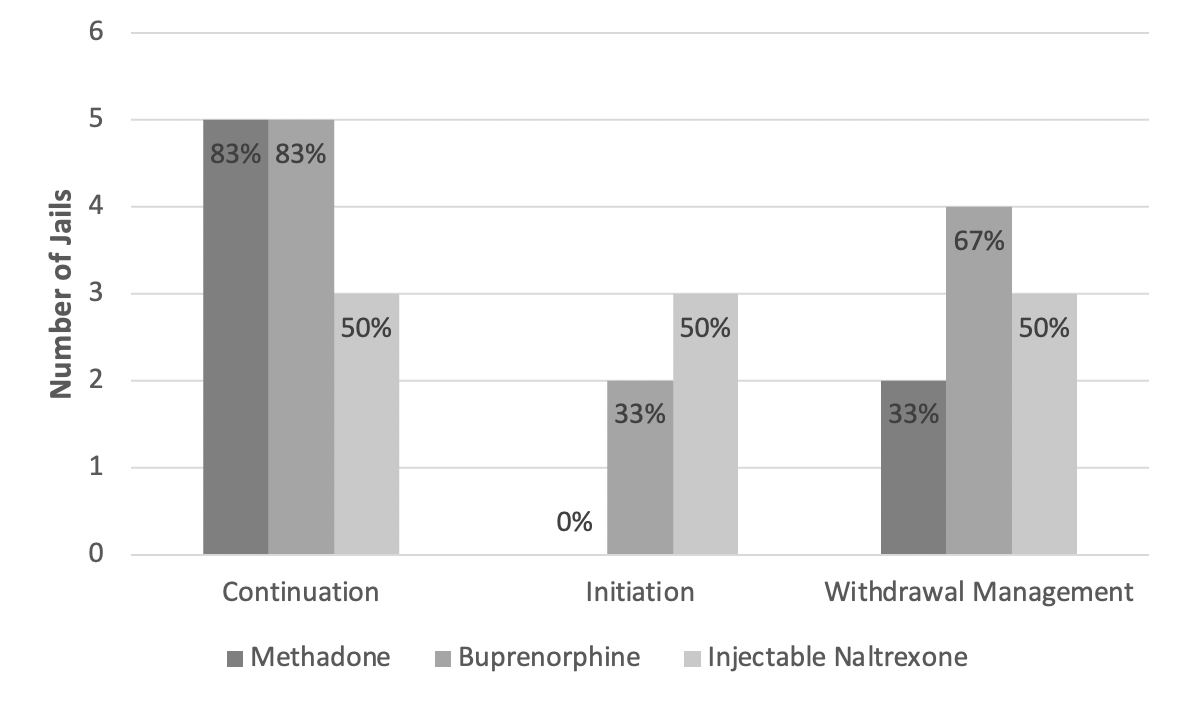
*
